# Application of AMOR in Craniofacial Rabbit Bone Bioengineering

**DOI:** 10.1155/2015/628769

**Published:** 2015-01-29

**Authors:** Marcelo Freire, Jeong-Ho Choi, Anthony Nguyen, Young Deok Chee, Joong-Ki Kook, Hyung-Keun You, Homayoun H. Zadeh

**Affiliations:** ^1^Department of Applied Oral Sciences, The Forsyth Institute, Cambridge, MA, USA; ^2^Department of Infection and Immunity, Harvard School of Dental Medicine, Boston, MA, USA; ^3^Department of Orthodontics, School of Dentistry, Seoul National University, Jongno-gu, Seoul 110-749, Republic of Korea; ^4^Ostrow School of Dentistry, University of Southern California, Los Angeles, CA, USA; ^5^Department of Periodontology, College of Dentistry, Wonkwang University, Iksan, Republic of Korea; ^6^Department of Oral Biochemistry, School of Dentistry, Chosun University, Gwangju, Republic of Korea; ^7^Laboratory for Immunoregulation & Tissue Engineering, Herman Ostrow School of Dentistry, University of Southern California, 925 West 34th Street, Los Angeles, CA 90089, USA

## Abstract

Endogenous molecular and cellular mediators modulate tissue repair and regeneration. We have recently described antibody mediated osseous regeneration (AMOR) as a novel strategy for bioengineering bone in rat calvarial defect. This entails application of anti-BMP-2 antibodies capable of* in vivo* capturing of endogenous osteogenic BMPs (BMP-2, BMP-4, and BMP-7). The present study sought to investigate the feasibility of AMOR in other animal models. To that end, we examined the efficacy of a panel of anti-BMP-2 monoclonal antibodies (mAbs) and a polyclonal Ab immobilized on absorbable collagen sponge (ACS) to mediate bone regeneration within rabbit calvarial critical size defects. After 6 weeks,* de novo* bone formation was demonstrated by micro-CT imaging, histology, and histomorphometric analysis. Only certain anti-BMP-2 mAb clones mediated significant* in vivo* bone regeneration, suggesting that the epitopes with which anti-BMP-2 mAbs react are critical to AMOR. Increased localization of BMP-2 protein and expression of osteocalcin were observed within defects, suggesting accumulation of endogenous BMP-2 and/or increased de novo expression of BMP-2 protein within sites undergoing bone repair by AMOR. Considering the ultimate objective of translation of this therapeutic strategy in humans, preclinical studies will be necessary to demonstrate the feasibility of AMOR in progressively larger animal models.

## 1. Introduction

Bioengineering strategies often use modulation of the extracellular environment to regulate cell fate and guide tissue differentiation. To date, tissue engineering approaches focus on either cells delivery to the tissue of interest, or scaffold-based delivery of signaling molecules to stimulate cell migration, differentiation, and regeneration [1–5]. Bone healing requires both resident cells and endogenous bioactive molecules that are locally produced or brought into the circulation to the extracellular matrix (ECM) to activate the cascade of repair [6–17].

Expression of bone morphogenetic proteins (BMPs) during bone repair is required for osteogenesis [18–20]. More specifically, endogenous BMP2 plays an essential role in initiating the early cascade of bone healing, ectopic bone formation, and adult ossification [21, 22]. Because of this intrinsic role, rhBMP2 has been utilized clinically for applications related to bone regeneration since FDA approval [23]. The use of exogenous delivery of these molecules has been reported to successfully regenerate bone for various clinical scenarios including spinal fusion, nonfracture union, and craniofacial applications [5, 24, 25].

Although recombinant human BMPs are the most studied growth factors for tissue repair clinically, controlled-release and protein engineering strategies have been recently reported to provide retention of endogenous growth factors within matrices [26–28]. Furthermore, recent evidence has shown that immobilized antibodies can perform the role of a complementary molecule to sequester endogenous BMP-2 and induce bone regeneration [27, 29]. Antibody mediated osseous regeneration (AMOR) was shown to be effective in rat calvaria critical size defect model, and it demonstrated that when defects are treated with anti-BMP-2 antibodies immobilized into absorbable collagen sponge (ACS), bone repair is completed after 6 weeks.

We therefore hypothesized that, in order to validate AMOR as a viable method of tissue engineering, it is important to demonstrate this phenomenon in multiple animal models. Here we tested* in vivo* the capability of the antibodies to promote bone regeneration in rabbit calvaria. Rabbit and human BMP-2 share high degree of homology of both nucleotide and protein sequence, supporting the feasibility of the presented animal model. Our results demonstrated that osteogenesis was activated when BMP-2 was bound to various antibody clones, including C6, C9, C19, C20, C22, 4B12, and 3G7. Consistent with previous results, anti-BMP-2 antibody clones C22 and 3G7 mediated significant bone regeneration* in vivo*. Our objective is to progressively use larger animal models and test AMOR preclinically, with direct implications for the translation of the proposed strategy in humans.

## 2. Materials and Methods

### 2.1. Antibodies

Antibodies utilized in this study, including monoclonal antibodies (mAbs) and polyclonal antibodies (pAbs), were previously listed. In brief, rhBMP2 (Infuse, Medtronic, TN) molecule was used as immunogen in mice for generation of mAbs. Hybridoma cloning kit, ClonaCell-HY (StemCell Technologies, Vancouver, BC, Canada), was used to generate clones (C3–C24) according to the manufacture's protocol. Isotype-matched control antibody against KLH was utilized as control antibody (R&D systems, Minneapolis, MN).

### 2.2. *In Vivo* Critical Size Defect Model

The animal procedures were reviewed and approved by the Institutional Animal Care and Use Committee (IACUC) of the University of Southern California. To investigate the ability of specific anti-BMP-2 Abs to mediate AMOR* in vivo*, the calvarial defect models in 3 adult New Zealand white rabbits were utilized. Eighteen calvarial defects were created in 12-week-old rabbits under general anesthesia using xylazine (20 mg/mL) and ketamine (10 mg/mL). Inhalation with isoflurane was maintained at 2–2.5% (ISofulorande, Buffalo). The flow rate for oxygen was 0.3 l/min and for NO_2_ 0.2 l/min. The scalps were shaved, and aseptic presurgical and operative procedures were followed according to the University Southern California guidelines. Full thickness skin flaps were raised and the left and right parietal bones were exposed. Eight mm diameter defects in parietal bones were generated using a trephine drill under copious saline irrigation. Various Ab molecules were immobilized on absorbable collagen sponge (ACS) by 1 hour incubation at room temperature. The periosteum was approximated with absorbable 5.0 PGA suture, followed by the skin, using 6.0 polypropylene sutures. Postoperative analgesics (Buprenorphine, 0.02 to 0.05 mg/kg bw) were administered twice a day for 3 days. At 45 days, animals were sedated with ketamine/xylazine and euthanized by an injection of Pentobarbital 120 mg/kg i.v and the calvarial bones were excised.

### 2.3. Microcomputed Tomography (* μCT) Analysis*

Six weeks following surgical procedure, animals were sacrificed. Each rabbit calvarial specimen was placed in the cranial-caudal direction in a sample holder and scanned using a high-resolution micro-CT system (MicroCAT II, Siemens Medical Solutions Molecular Imaging, Knoxville, TN). The spatial resolution of the scanned image was 43.743 *μ*m (Voxel dimension), and bit depth was 16 bits. After scanning, the 2D image data was stored in the Digital Imaging and Communications in Medicine (DICOM) format, and transferred to a computer, where a 3D reconstruction and analysis were performed. Each slice image in DICOM format was so big in file size as about 3 MB; therefore reduction of the size of data was inevitable for computation. Calvarial region was cropped and saved from the obtained consecutive microtomographic slice images as a volume of interest (VOI) using Amira software (Visage Imaging, San Diego, CA), in order to reduce the size of data. In this step, the original spatial resolution and bit depth were maintained and there was no specific data loss, because data were not resampled. The volume of new bone in calvarial defect was measured using V-Works 4.0 software (Cybermed Inc., Seoul, Korea). The skeletal tissue was segmented using a global thresholding procedure. New bone was separated from preexisting bone by applying a cylindrical divider whose base is same as the defect, and the volume (mm^3^) was calculated [27, 29, 30].

### 2.4. Histology

Bone specimens were fixed with 10% neutral buffered formalin (Richard-Allan Scientific, Kalamazoo, MI) for 48 h at room temperature, followed by decalcification in standard decalcifying solution (Richard-Allan Scientific) for 48 h at 4°C. Tissues were sequentially dehydrated in graded alcohol (30%–100%) and paraffin embedded. Sectioned (6 *μ*m) samples were stained with Hematoxylin and Eosin (H&E) and Masson Trichrome blue (Sigma) for morphology evaluation.

### 2.5. Histomorphometric Analysis

Quantitative analysis of bone regeneration was conducted by applying standard histomorphometric techniques. Measurements were carried out on region of interest of 16x mag images and analysis utilized NIH/Scion Image J (Scion corp.). Newly osteoid bone was quantified and expressed as percentage (%) and compared to isotype-matched control antibody. ASBMR methods and nomenclature were used in quantification of new bone formed [31].

### 2.6. Immunostaining

Rabbit calvarial bone sections were rehydrated and washed three times with 1X PBS. They were blocked with normal horse serum (Vector laboratories, CA) and incubated at 4°C overnight with a polyclonal IgG antibody against either BMP-2 (Rabbit, Biovision, CA) or Osteocalcin (Rabbit, Genway, CA), diluted in PBS at 1 : 500 and 1 : 30, respectively. Slides were incubated in isotype-matched control Abs or PBS without primary Abs as negative controls. The antibody-treated and negative control sample slides were washed with PBS and incubated with horseradish peroxidase (HRP)-conjugated goat anti-rabbit IgG Abs (Invitrogen, CA) diluted at 1 : 50 in PBS at 37°C for 30 min. HRP substrate was used for visualization and sections were then counterstained with Mayer's Hematoxylin (Vector Laboratories, CA).

### 2.7. Statistics

The Student's* t*-test was used for pairwise comparisons as indicated. Statistical significance was assigned at *P* < 0.05.

## 3. Results

### 3.1. Anti-BMP-2 Antibodies Mediated* In Vivo* ****Repair of Critical-Sized Rabbit Calvarial Defects

**Figure 1:**
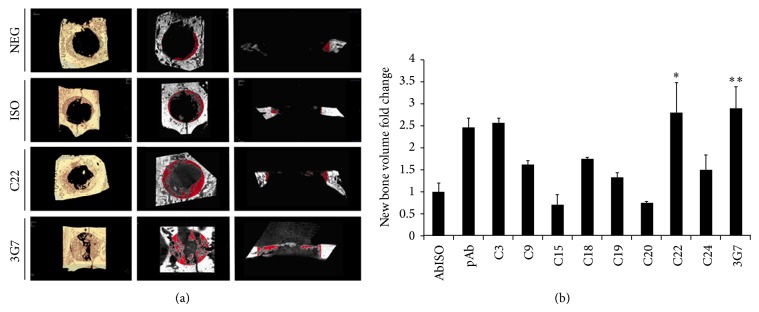
Micro-CT analysis of* de novo* bone formation within rabbit calvarial defects. Three-dimensional micro-CT images of bone specimens after 6 weeks of surgical creation of critical size defect (a). Representative sample of absorbable collagen sponge treated with plain PBS (Neg) or with immobilized antibodies. Antibodies included isotype matched control mAb (Iso), polyclonal anti-BMP-2 Ab (pAb), or BMP-2-specific mAb clones (C3, C15, C18, C20, C22, C24, and 3G7). Quantitative *μ*CT of new bone volume (mm^3^) of respective controls and anti-BMP-2 clones are shown (b). Red highlighted area demarcates segmented volume corresponding to the area of the original defect. This highlighted area represents the volume which was measured during quantitative analysis. Results are presented as fold change and ± SD versus isotype antibody control (*n* = 3 for Neg, ISO, C22, 3G7 samples only). ^*^ *P* < 0.05 or ^**^ *P* < 0.01.

The ability of anti-BMP-2 antibodies to accelerate* in vivo* bone regeneration and repair in rabbits was for the first time investigated (Figure 1). A panel of anti-BMP-2 antibodies immobilized on absorbable collagen sponge was implanted within critical-sized calvarial defect in parietal bone of rabbits. The Abs used included anti-BMP-2 monoclonal and polyclonal Abs, as well as isotype-matched control Abs. After 6 weeks, animals were euthanized and specimens were collected. Micro-CT analysis of calvarial bones implanted with immobilized C22 and 3G7 antibodies demonstrated increased bone deposition. The volume of these newly formed bone fills was statistically significant when compared with isotype-matched control antibodies. In contrast, control treatment, including ACS alone or isotype control antibodies, did not present any degree of calvaria bone repair during the experimental period. To be able to screen a large number of antibody clones in rabbits, only some of the immobilized antibodies were implanted in triplicates, allowing statistical comparison (isotype control Ab, C22 monoclonal antibody, and anti-BMP-2 Ab, 3G7 anti-BMP-2 Ab). Statistical measurement was only possible in the samples with triplicates; the remaining groups were tested in duplicates or just as initial screening information regarding their potential to induce AMOR* in vivo* in rabbits. In order to gauge the biological significance of those Abs with potential ability to mediate bone repair, when statistical analysis was not possible, those with numeric values that were more than two standard deviations above mean of isotype control Ab were considered as potentially osteogenic. Representative three-dimensional volume reconstructions are shown (Figure 1(a)). Quantitative analysis of new bone volume fold change to isotype control demonstrates higher density when calvarial defects are treated with pAb, C3, C22, and 3G7 (Figure 1(b)). These findings support the evidence that increase bone regeneration* in vivo.*

**Figure 2:**
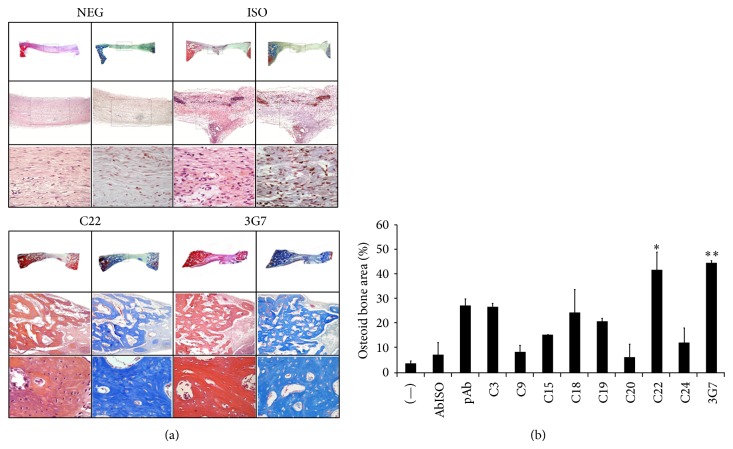
Bone tissue healing is evaluated by histological and Histomorphometric analysis. Critical size 8 mm bone defects were created in rabbit calvaria, which were implanted with collagen sponge alone (NEG) or with immobilized antibodies and sacrificed at 6 weeks. The antibodies tested were isotype-matched control Ab (Iso), polyclonal Ab (pAb), or BMP-2-specific Ab clones (C3, C15, C18, C20, C22, C24, and 3G7). H&E (left panels) and Trichrome blue staining (right panels) were performed (a). Histomicrographs are presented in low and high magnifications (16x, 100x, and 400x). Newly formed bone is embedded with osteocytes (black arrows, a) and quantified by histomorphometry (b). Percentage of osteoid bone area (mm^2^) demonstrated significant bone deposition in the defect region when treated with C22 and 3G7 anti-BMP-2 mAb clones. Means and standard deviations of replicate groups are shown (Neg, ISO, C22, 3G7). ^*^ *P* < 0.05 or ^**^ *P* < 0.01, versus isotype control.

To confirm the nature of radio-dense material presented in micro*-*CT results, bone across the region of the defect was characterized by histological analysis. At 6 weeks, harvested tissues were fixed, paraffin-embedded, and sectioned. Histological staining with H&E and Trichrome blue was performed. Histomicrographs of calvarial defects are representative of samples implanted with either untreated collagen sponge or immobilized antibodies (isotype, C22, 3G anti-BMP-2 mAbs) (Figure 2(a)). After 6 weeks, control defects formed primarily connective tissue without any apparent bone fill. In contrast, implantation of immobilized C22 and 3G7 resulted in significant bone formation within calvarial defects (Figure 2(a)).

Histomorphometric analysis performed on sections at 6 weeks confirmed the qualitative results observed (Figure 2(b)). New bone fill in each of the defects was expressed as osteoid bone within the region of interest. Consistently, results demonstrated statistically significant higher degree of bone repair when defects were implanted with 3G7 or C22 anti-BMP-2 mAbs compared with isotype controls (Figure 2(b)).

Careful examination of all calvarial sections receiving antibodies immobilized on collagen sponge did not reveal any adverse responses, such as inflammatory infiltrate or tissue damage. Postsurgery clinical features of animals, as well as histological analysis, demonstrated normal recovery and tissue regeneration when antibodies were utilized.

### 3.2. Detection of* In Situ* BMP-2 and Osteocalcin Protein Expression within Treated Sites

**Figure 3:**
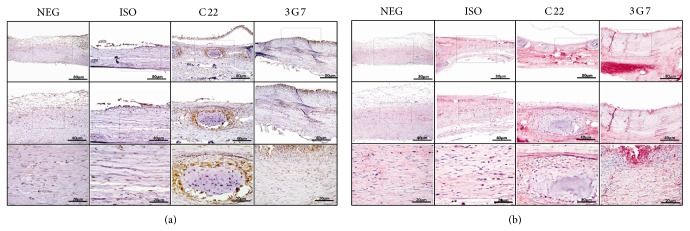
Immunostaining reveals* in situ* BMP-2 and Osteocalcin protein expression. Rabbit calvaria surgical defect was treated with similar described treatments. Sections were labeled with anti-BMP-2 polyclonal Ab (a panel, brown), or anti-Osteocalcin polyclonal Ab (b panel, red) followed by HRP-conjugated secondary Ab. Immunohistochemistry revealed increased BMP-2 (a) and Osteocalcin (b) expression, in sites implanted with anti-BMP-2 mAb clones C22 and 3G7 at 6 weeks. Positive immunostaining of BMP-2 and Osteocalcin is indicated by black arrow. Negative staining for both molecules is represented by X symbol.

To characterize the healing of defects treated with various Abs, the expressions of specific markers of osteogenesis, namely, BMP-2 and Osteocalcin, were examined. Rabbit calvarial defects implanted with ACS-Ab were immunolabeled with polyclonal anti-BMP-2 or anti-Osteocalcin antibodies as the primary Abs to detect the* in situ* expression of the molecules to which these Abs bind (Figure 3). High intensity of BMP-2 expression was detected in sites implanted with anti-BMP2 mAb clones (C22,3G7), confirming past results [27]. Ossification centers exhibited intense BMP-2 protein expression. Lower BMP-2 labeling was shown in calvarial defects, which were implanted with collagen sponges alone or with isotype controls. The* in situ* BMP-2 expression correlated well with increased bone regeneration.

Osteocalcin expression is presented as a late marker of osteogenesis and consistent with mature bone. To investigate Osteocalcin protein expression in the tissue, additional immunostaining was performed. Intense Osteocalcin expression was observed within calvarial defects treated with C22 and 3G7 clones, compared to isotype-matched control Abs, while baseline labeling was presented with immobilized isotype-matched antibody and negative controls. These findings support the notion that the presence of the osteogenic anti-BMP-2 Abs favors the expression of BMP-2, providing evidence for the mechanism of action of AMOR, involving accumulation of endogenous BMPs in sites of anti-BMP-2 Ab implantation. This in turn mediates osteogenesis, which involves local expression of BMPs, thus amplifying the BMP signal.

## 4. Discussion

The hypothesis pursued in the present study has been that bone formation by AMOR is attributable to the ability of anti-BMP-2 mAbs to capture and tether endogenous BMP-2 in a biologically active orientation. We continue to test this hypothesis in different animal models and the efficacy of immobilized anti-BMP-2 mAb to regenerate rabbit bone tissue. This report demonstrates an effective screening method for a panel of antibodies* in vivo* and the mechanism by which the tissue healing acceleration occurs. The significance of the need to pursue multiple animal models is because most of the anti-BMP2 antibodies used here were generated in murine host against human BMP2 immunogen. The fact that these antibodies were able to induce bone repair in rabbits suggests that the antibodies likely cross-reacted with rabbit BMPs. This may be attributed to the significant degree of homology between human, rat, rabbit, and murine BMP-2 proteins [6, 12, 29, 32]. In our analysis, the sequences of BMP-2 were aligned and the degree of similarity was calculated. The GenBank accession numbers of human and rabbit BMP2s were KC294426 and NM_001082650, respectively. CLUSTAL W algorithm in the MegAlign program (DNAStar Lasergene 8.0, DNAStar Inc., USA) was used. The similarity between human and rabbit BMP2s in DNA and protein levels was 90.7% and 95.4%, respectively (Supplementary Figure 1 in Supplementary Material available online at http://dx.doi.org/10.1155/2015/628769).

Therapeutic antibodies are used extensively in clinical practice with excellent efficacy and safety track record [33–35]. Most of the current clinical applications of therapeutic antibodies involve repeated systemic administration for treatment of chronic diseases or multiple applications for cancer therapy, which entail relatively high doses of antibodies. Our proposed therapeutic approach is to implant mAb immobilized on a scaffold in a local site as a single therapy. Here, we took advantage of the efficacy of antibodies to tether endogenous BMP-2 and accelerate osteogenesis in rabbit calvaria. The full closure of the defect was not observed, most likely because healing of rabbit calvarial critical size defects takes 8–12 weeks [7, 36]. The 6 weeks timepoint selected in the present study made the expedited comparison of bone healing within experimental and control sites possible.

Recent reports have uncovered serious safety concerns with rhBMP-2 therapy, documenting much higher incidence of adverse reactions than previously revealed in industry-sponsored studies [37]. In 2011, the database of the Manufacture and User Facility Device Experience contained 83 reports of adverse events after oral and maxillofacial operations involving implantation of exogenous rhBMP-2 [38]. After orthopedic applications utilizing rhBMP-2, an estimate of adverse events of 10% to 50% has been suggested, depending on the approach. In anterior cervical fusion with rhBMP-2 has an estimated 40% greater risk of adverse events with rhBMP-2 in the early postoperative period, including life-threatening events. After anterior interbody lumbar fusion, rates of implant displacement, subsidence, infection, urogenital events, and retrograde ejaculation are higher when rhBMP-2 is administered compared with controls [39]. Posterior lumbar interbody fusion use has been reported to be associated with radiculitis, ectopic bone formation, osteolysis, and poorer global outcomes [40, 41]. In posterolateral fusions, the risk of adverse effects associated with rhBMP-2 is equivalent to or greater than that of iliac crest bone graft harvesting, where 15–20% of subjects report early back pain and leg pain adverse events [40, 42]. Higher doses of rhBMP-2 are also associated with a greater apparent risk of new malignancy [40].

In our study, adverse events have not been detected, since endogenous BMPs are captured, in contrast to supraphysiologic doses of exogenous rhBMP-2 required to be administered in exogenous growth factor tissue regeneration approach [38, 40, 43]. Our previous studies have also not observed any adverse events in other models [27, 29, 44]. Qualitative differences were found between the nature of the bone regenerated in sites treated with rhBMP-2 and those with anti-BMP-2 mAb [29]. In this study, the early radiopaque formation seen in micro-CT results was coincident with increased endogenous BMP-2 detection by immunohistochemistry (Figure 3). This is in agreement with our previous results that demonstrated increased local BMPs expression [27, 29].

Adverse reactions encountered with mAb therapy include acute anaphylactic (IgE-mediated), serum sickness, tumor lysis syndrome, and cytokine release syndrome [45]. The clinical manifestation can range from local skin reactions at the injection site, pyrexia, and influenza-like syndrome, to acute anaphylaxis and systemic inflammatory response syndrome. However, comparatively, therapeutic antibodies have fewer adverse reactions than other drugs. This is evidenced by much higher clinical approval rate of 20% for therapeutic antibodies by FDA, as compared with 5% for conventional drugs [46]. Therapeutic monoclonal antibodies used locally in low concentrations have relatively low incidence of adverse reactions [34, 47]. In our study, local administration of anti-BMP-2 antibodies (160 nM) on collagen sponge is substantially less than high concentrations of systemic application of antibodies and no observed side effects were manifested.

It has been demonstrated that the efficiency of exogenous rhBMP-2 is approximately 10-fold lower when compared to endogenous BMP-2 in the formation of* de novo* bone in ectopic sites [48]. We demonstrate that, by tethering endogenous BMP-2, acceleration of healing is mediated and consequently bone regeneration occurs [27, 29, 44]. In contrast, the effective clinical dose of exogenous rhBMP-2 (1.5 mg/mL) is several orders of magnitude higher than the physiologic concentrations of BMP-2 [15]. In regard to half-life, antibodies have long half-life (30–100 days), whereas exogenous recombinant BMP-2 exhibits short half-life (7–16 min systemically and up to 2 days with carrier). Because of increased half-life, lower concentrations of antibodies are sufficient to achieve therapeutic efficacy, decreasing the risk of side effects. This notion was firstly supported by the identification that locally produced BMPs in the microenvironment had high local concentrations for few days when surgical sites were treated with therapeutic antibodies [29] and later this same approach confirmed that this persistence was longer than 30 days [44].

The present report confirms that anti-BMP-2 Abs immobilized on a solid scaffold are able to bind to endogenous BMP-2 and accelerate bone healing. For the first time, the efficacy of the antibodies of AMOR was shown in rabbit bone. This translational approach has excellent reproducibility in previous and current animal models and holds promising results for future clinical trials.

## Supplementary Material

Supplementary Figure S1. Protein homology between human and rabbit BMP-2. The primary amino acid sequences of human and rabbit BMP-2 were aligned and calculated the similarity of them using the CLUSTAL W algorithm in the MegAlign program (DNAStar LasergeneTM 8.0, DNAStar Inc., Madison, WI, USA). Pairwise comparison is represented in similarity percentage (95.4%).
