# Supplementary figures and images for: Application of AMOR in Craniofacial Rabbit Bone Bioengineering
Source: Biomed Res Int. 2015 Jan 29;2015:628769. doi: 10.1155/2015/628769 (PMC4325208; doi:10.1155/2015/628769)

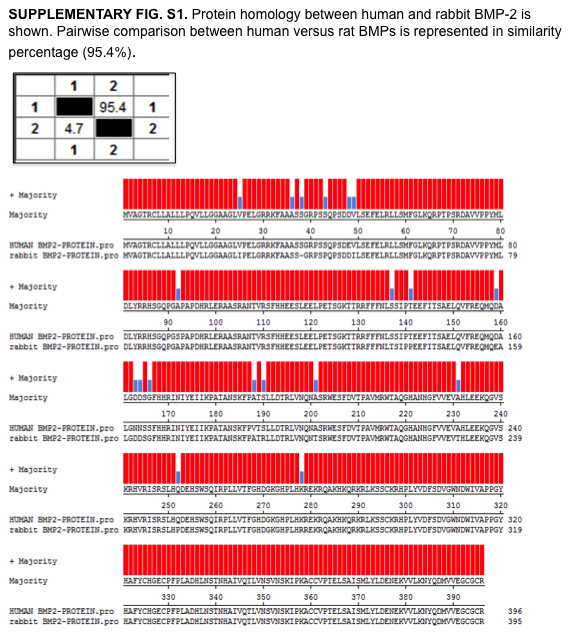

Supplement: Supplementary file 1 — Supplementary Figure S1. Protein homology between human and rabbit BMP-2. The primary amino acid sequences of human and rabbit BMP-2 were aligned and calculated the similarity of them using the CLUSTAL W algorithm in the MegAlign program (DNAStar LasergeneTM 8.0, DNAStar Inc., Madison, WI, USA). Pairwise comparison is represented in similarity percentage (95.4%). [file 628769.f1.docx]
